# Characterization of the atypical lymphocytes in African swine fever

**DOI:** 10.14202/vetworld.2016.792-800

**Published:** 2016-07-30

**Authors:** Z. A. Karalyan, Z. R. Ter-Pogossyan, L. O. Abroyan, L. H. Hakobyan, A. S. Avetisyan, N. Yu Karalyan, E. M. Karalova

**Affiliations:** Laboratory of Cell Biology and Virology, Institute of Molecular Biology of NAS, 7 Hasratyan St, 0014 Yerevan, Armenia

**Keywords:** African swine fever virus, atypical lymphocytes, CD2, DNA ploidy

## Abstract

**Aim:**

Atypical lymphocytes usually described as lymphocytes with altered shape, increased DNA amount, and larger size. For analysis of cause of genesis and source of atypical lymphocytes during African swine fever virus (ASFV) infection, bone marrow, peripheral blood, and *in vitro* model were investigated.

**Materials and Methods:**

Atypical lymphocytes under the influence of ASFV were studied for morphologic, cytophotometric, and membrane surface marker characteristics and were used *in vivo* and *in vitro* models.

**Results:**

This study indicated the increased size, high metabolic activity, and the presence of additional DNA amount in atypical lymphocytes caused by ASFV infection. Furthermore, in atypical lymphocytes, nuclear-cytoplasmic ratio usually decreased, compared to normal lymphocytes. In morphology, they looking like lymphocytes transformed into blasts by exposure to mitogens or antigens *in vitro*. They vary in morphologic detail, but most of them are CD2 positive.

**Conclusions:**

Our data suggest that atypical lymphocytes may represent an unusual and specific cellular response to ASFV infection.

## Introduction

The atypical lymphocyte is a non-malignant abnormal leukocyte seen in the peripheral blood. It produced in a variety of disorders and usually arises as a non-specific response to stress from a variety of stimuli. A lymphocyte becomes larger in size and sometimes capable of dividing. The cells vary greatly in size and shape. It was originally described by Türk in the peripheral blood of patients with infectious mononucleosis [1]. First classification and detailed description of atypical lymphocytes were done in patients with the same disease in 1923. At this time, Downey and McKinlay gave a complete description of the pathological mononuclear cells present in large numbers in this disease. They divided these cells into three types, classified since then as Downey cells Type I, II, and III, and now referred to as atypical lymphocytes or virocytes [1]. Morphology of atypical lymphocytes may differ from one case to another. However, these lymphocytes can be identified by their unusual morphology, increased size, and presence of active DNA synthesis [1]. Atypical lymphocytes appear not only in infectious mononucleosis but also at a variety of other viral diseases [2,3].

In our previous reports, peripheral blood lymphocytes containing abnormalities in the nuclear structure and DNA amount have been described [4]. These abnormalities were noted in lymphocytes in pigs with acute African swine fever (ASF). This report describes the origin, morphology, and cytochemistry of the atypical lymphocytes in pigs with acute ASF.

For analysis of cause of genesis and source of atypical lymphocytes during ASF virus (ASFV) bone marrow (BM), peripheral blood, in *in vivo* model and primary culture of BM cells (PCBM) in *in vitro* model were investigated atypical cells in *in vivo* and *in vitro* conditions.

Therefore, the present study was carried out to detect main phenotypical and morphological characteristics of ASFV derived atypical lymphocytes.

## Materials and Methods

### Ethical approval

All procedures of sampling collection were performed strictly as specified by Independent Ethics Committee of the Institute of Molecular Biology of NAS, IRB00004079 with minimal stress to animals.

### Virus

Infections were carried out using ASFV (genotype II) distributed in Republic of Armenia and Republic of Georgia. The titer of ASFV for each intramuscular injection was 10^4^ hemadsorbing doses (HAD50)/ml. Virus titration was done as described previously and expressed as log10 HAD50/ml for non-adapted cells [4].

### Animals

Animal care and euthanasia were done according to the AVMA Guidelines on Euthanasia, and local guideline for animal care and use. Carbon dioxide inhalation (75-80% carbon dioxide for 20 min) was used to euthanatize animals. The series of investigations here presented are based on examination of the BM obtained from six piglets (3 months) beings.

### PCBM

The porcine PCBM was obtained from the femoral bones of six healthy, 6-8 weeks old piglets [5]. The initial amount of the cells we used for each experiment were 10^6^ cells/ml. Cells were cultured in the RPMI 1640 supplemented with 10% fetal bovine serum, L-glutamine, and 2% penicillin-streptomycin, at 37°C incubator in the atmosphere of CO_2_ (3). For experiments, cells were examined at 24, 48, 72, and 96 h post cultivation.

### Analysis of the white blood cells

Blood, BM, and PCBM slides were stained with Giemsa (Sigma–Aldrich) in a 1:5 dilution in water and examined under the light microscope at 1000× in a random sequence without knowledge of the day of ASF disease to the investigator. At least, 200 white blood cells in each sample were identified and classified. The identification of cells and their sizes was done as described previously [4,5].

### Cytometry

Measurements were performed by the scanning image analyzer (magnification 12.5 × 100). The evaluation of cell sizes was done by routine cytometry using ImageJ software. The nuclear-cytoplasmic (N: C) ratio was measured as a ratio of the size of the nucleus of a cell to the size of the cytoplasm of that cell.

### Cell sorting

Atypical cells were separated from peripheral blood mononucleated cells. CD2+ cells were captured by an antihuman immunoglobulin G (IgG) monoclonal antibodies (ABs) conjugated to magnetic beads (Dynal AS, Oslo, Norway, 11159D). Magnetic bead-bound cells were separated on a magnetic particle concentrator (Dynal MPC- 6R, Dynal AS, Oslo, Norway). CD2 mononuclear cells were isolated with human IgG MAb conjugated to magnetic beads (Dynal Biotech ASA, Oslo, Norway). Briefly, cells were washed and resuspended in phosphate buffer saline, supplemented with 2% fetal calf serum (Gibco), and incubated with Dynabeads CD2 (11159D) as described in the protocol. Samples were placed into the MPC, and the suspended cells were discarded. Bound cells were treated with Dynabeads cell separation guide as described in protocol. After magnetic cell separation, we further used microscopy to analyze the sorted cells.

### Ploidy of cells

DNA content was expressed on a “c” scale, in which 1 c is the haploid amount of nuclear DNA occurred in normal (non-pathologic) diploid populations in G0/G1. The DNA content of unstimulated swine lymphocytes was used as a diploid standard for measurements. DNA measurements identify nuclei as aneuploid if they deviate more than 10% from 2 c, 4 c, 8 c, or 16 c, i.e., if they are outside of 2 c±0.2, 4 c±0.4, 8 c±0.8, or 16 c±1.6 values. The total number of cells in euploid areas of the DNA histogram rescaled by the mean corrective factor (1.8 c-2.2 c, 3.6 c-4.4 c, 7.2 c-8.8 c, and 14.4 c-17.6 c) was also calculated.

The variability of DNA content in unstimulated lymphocytes did not exceed 10%.

### RNA cytophotometry

The total RNA content in all investigated cells was measured by the method of cytophotometry on the image analyzer (Opton-05) under such conditions: Objective 100×, wavelength 620 nm, scanning step 0.2 µm. Smears were stained by gallocyanin-chrome alum [6].

### DNA protein staining

All preparations were treated with the combined Feulgen-Naphthol yellow staining procedure [7]. This method permits simultaneous microspectrophotometric analyses of DNA and protein in single cells, and the protein value is closely correlated to the amount of dry mass of the cell. Also was used to Feulgen stained cell identification.

### Statistical analysis

All *in vitro* experiments were conducted in triplicate. The significance of virus-induced changes was evaluated by two-tailed Student’s t-test. p<0.05 were considered significant. SPSS version 17.0 software package (SPSS Inc., Chicago, IL, USA) was used for statistical analyses.

## Results

### Atypical lymphocytes morphology and cytometry

**Figure-1:**
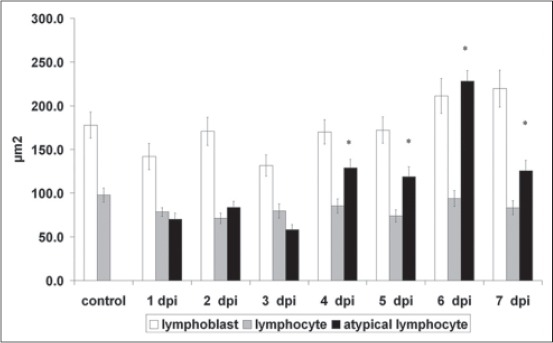
Size of lymphoid cells in the peripheral blood of African swine fever virus-infected pigs. X axis is the days post infection (dpi). *Significant compared with lymphocytes.

Pigs with acute ASF showed highly variable white blood cell and particularly lymphocyte counts [4]. Lymphocytes from smears were evaluated morphologically based on size and NC characteristics as normal, reactive (increased overall size and cytoplasm), and atypical with increased size with cleaved nuclei. The same data were obtained from virus-infected PCBM (Figure-1).

**Figure-2:**
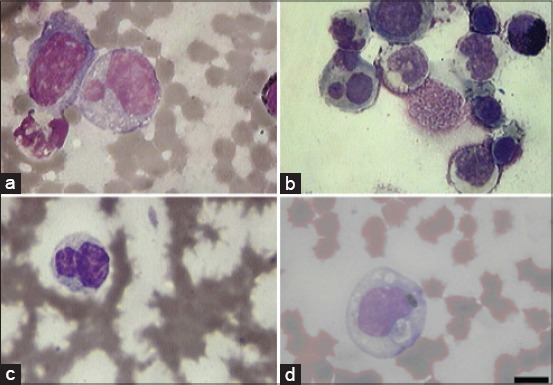
Arising of atypical lymphocyte under the influence of African swine fever virus (ASFV). (a) Atypical lymphocyte in bone marrow, (b) atypical lymphocyte in peripheral blood mononuclear cells, (c, d) atypical lymphocyte in peripheral blood of ASFV-infected pigs. Scale bare is 10 µm.

Atypical lymphocytes usually were medium- and big-sized with faintly basophilic cytoplasm (Figure-2). The nucleus of the atypical lymphocyte is usually few larger than that of the lymphocyte and is more irregular in shape. Internuclear bridges were absent; the cells had dense chromatin, in few cells were present readily visible nucleolus (usually single). Some other lymphocytes resembled like lymphoblast (observed separately) having more abundant cytoplasm and nucleoli (often two and sometimes more). The majority of the normal lymphocytes were small or medium showing a narrow rim of cytoplasm and the nuclear chromatin was dense without nucleoli.

Usually, the N:C ratio indicates the physiological state of lymphoid cells because in reactive lymphocytes the size of cytoplasm generally increases. In atypical lymphocytes, NC ratio also usually decreased, compared to normal lymphocytes (Table-1).

**Table-1:** The nuclear-cytoplasmic ratio of lymphocytes, lymphoblasts, and atypical lymphocyte in PCBM cells, blood, and bone marrow.

Cell localization	Groups	N/C ratio lymphocyte	N/C ratio lymphoblast	N/C ratio atypical lymphocyte
PCBM	24 h control	3.4±0.2	2.6±0.3	1.2±0.1*
	24 h ASFV	3.8±0.2	2.2±0.3	
	48 control	2.6±0.1	3.2±0.3	1.9±0.2*
	48 h ASFV	2.4±0.2	2.5±0.3	
	72 h control	2.5±0.2	2.2±0.2	1.3±0.2*
	72 h ASFV	2.6±0.1	2.6±0.2	
	96 h control	3.8±0.3	1.6±0.2	2.3±0.3
	96 h ASFV	1.6±0.2	2.9±0.2	
Blood (dpi)	Control	3.5±0.3		
	1	2.5±0.3	2.8±0.3	2.1±0.3
	2	2.4±0.2	3.0±0.3	1.9±0.1
	3	2.6±0.3	3.9±0.5	1.8±0.1*
	4	1.9±0.2	3.3±0.3	1.9±0.1
	5	2.0±0.2	3.2±0.3	2.4±0.2
	6	2.2±0.3	3.0±0.2	2.2±0.2
	7	2.9±0.3	2.7±0.2	2.3±0.2
Bone marrow (dpi)	Control	3.6±0.4	1.1±0.1	
	2	3.6±0.4	0.9±0.1	1.4±0.2*
	3	2.7±0.3	1.0±0.2	1.2±0.1*
	5	3.8±0.3	1.1±0.2	1.4±0.2*
	7	3.6±0.4	1.1±0.1	1.6±0.2*

* Significant compared to lymphocytes (p<0.05-0.01). ASFV=African swine fever virus, N/C=Nuclear-cytoplasmic, PCBM=Primary culture of bone marrow

### CD2 cells selection using monoclonal AB-coated magnetic beads

Lymphocyte subpopulations can be purified based on their cell-surface display of specific distinguishing molecules that can be recognized by monoclonal AB. AB against human and swine CD2 expressed high cross-reactivity [8], so were used human IgG MAb conjugated to magnetic beads.

We recognize that the majority of the atypical cells were CD2 positive.

**Figure-3:**
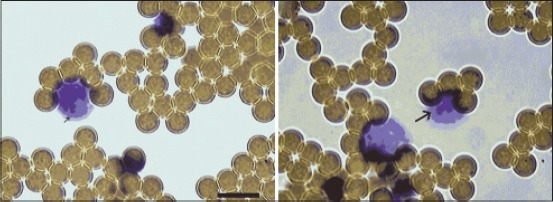
The target cells (peripheral blood mononucleated cells) bound to the CD2 beads. Arrows show CD2 magnetic beads-bound atypical lymphocytes. Scale bare is 10 µm.

It is good known that in swine there is a high frequency of CD4+/CD8+ dual expressing CD2+ T-cells. These cells have been reported to represent up to 60% peripheral T-cells [9]. This resulted in the isolation of a homogeneous population of swine CD2+ cells. Figure-3 shows the view of CD2+ magnetic bead coated atypical cells. Our data showed that majority of atypical cells carry CD2 phenotype.

### DNA ploidy index

**Figure-4:**
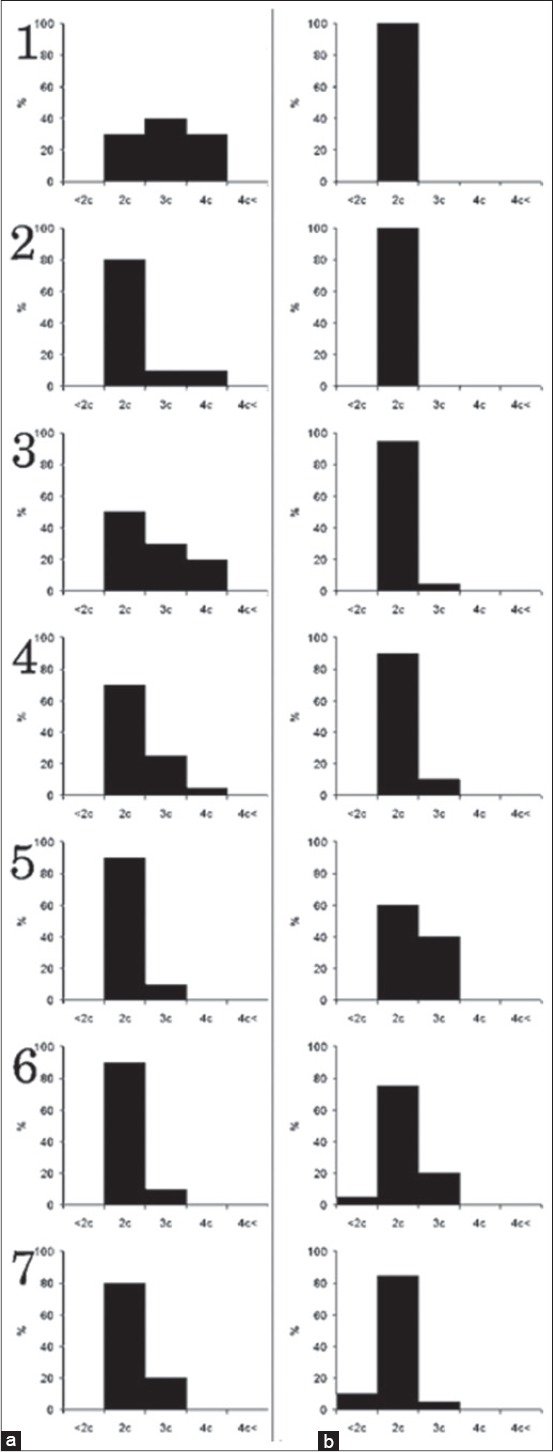
DNA ploidy blood cells. Columns: (a) Lymphoblasts, (b) atypical lymphocytes. Rows: 1 - 1 dpi; 2 - 2 dpi; 3 - 3 dpi; 4 - 4 dpi; 5 - 5 dpi; 6 - 6 dpi; 7 - 7 dpi; X-axis is the ploidy of cells. Y-axis is the percent of distribution of cells.

From the first days of disease in blood appear lymphoblasts, the population of lymphoblasts consists from diploid, hyperdiploid, and tetraploid cells (Figure-4a). During the progress of disease, histogram of distribution of nuclei by ploidy classes shifts to the left and at the terminal stage main amount of lymphoblasts is diploid and only 20% is hyperdiploid (Figure-4a). Atypical lymphocytes in blood appear only in pigs with ASFV infection. They are characterized by a shift of histogram to the right, and appearance of hyper tetraploid cells on 2 dpi. Then, histogram shifts to left (Figure-4b).

**Figure-5:**
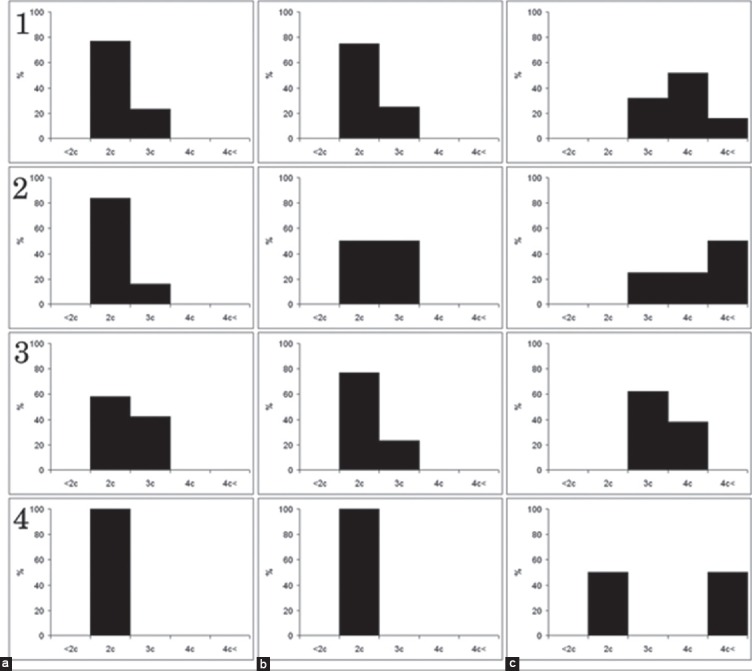
DNA ploidy bone marrow. Columns: (a) Lymphocytes, (b) atypical lymphocytes, (c) lymphoblasts. Rows: 1 - 2 dpi; 2 - 3 dpi; 3 - 5 dpi; 4 - 7 dpi; X-axis is the ploidy of cells. Y-axis is the percent of distribution of cells.

The distribution of the lymphocytes, lymphoblasts, and atypical lymphocytes in BM by DNA ploidy distribution is shown in Figure-5. In BM lymphocytes are diploid, but after ASFV infection, appear some cells with hyperdiploid nuclei (Figure-5a). Atypical lymphocytes in BM appear only in pigs with ASFV infection since 1 dpi. The main population of atypical lymphocytes in BM contained diploid and hyperdiploid DNA amount (Figure-5b) and kept till the end of disease. At first days of disease, BM lymphoblasts were shown by diploid, hyperdiploid, and tetraploid cells (Figure-5c). Distribution of lymphoblasts nuclei by ploidy classes does not demonstrate significant changes until the end of the disease.

**Figure-6:**
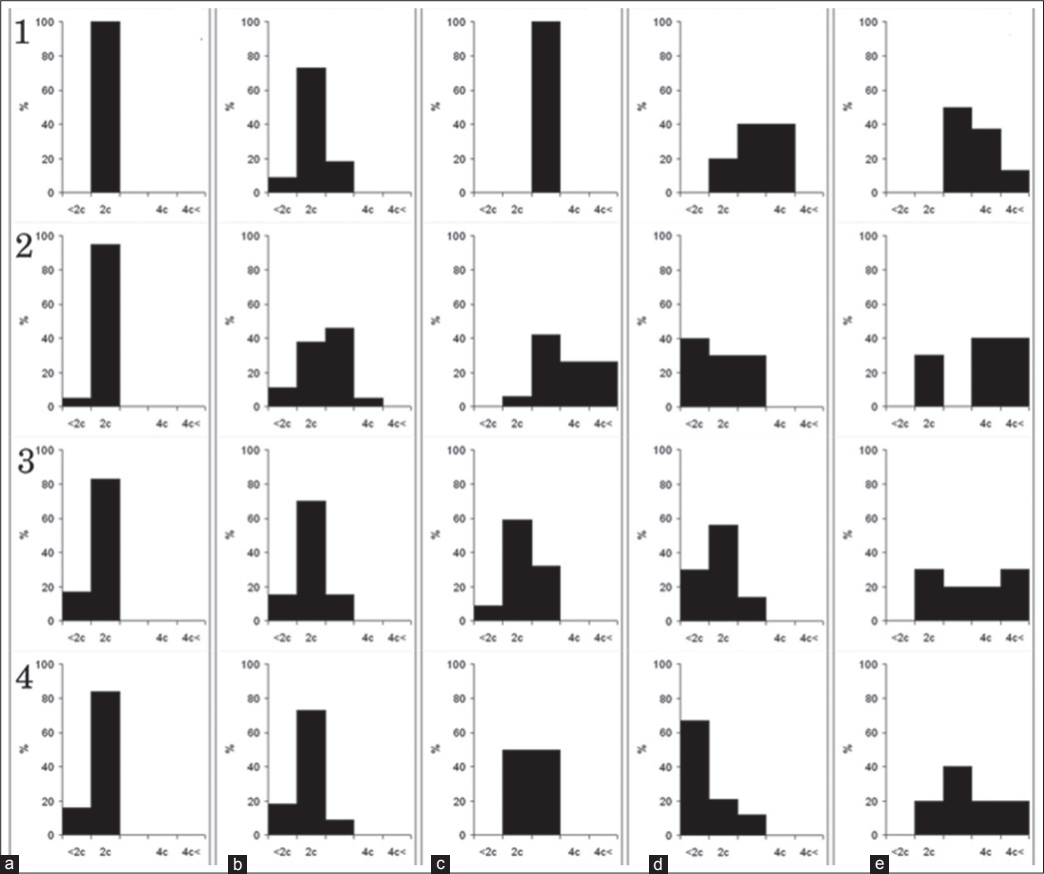
DNA ploidy bone marrow culture. Columns: (a) Control lymphocytes, (b) lymphocytes at acute African swine fever (ASF), (c) atypical lymphocytes at acute ASF, (d) control lymphoblasts, (e) lymphoblasts at acute ASF. Rows: 1 - 24 dpi; 2 - 48 dpi; 3 - 72 dpi; 4 - 96 dpi; X-axis is the ploidy of cells. Y-axis is the percent of distribution of cells.

In intact, PCBM lymphocytes are diploid. During the cultivation, histogram of the distribution of nuclei by ploidy classes shifts to the left and hypodiploid population appears (Figure-6a). In acute ASFV since 1 dpi appear cells with hyperdiploid and tetraploid nuclei (Figure-6b). In PCBM, atypical lymphocytes appear after 24 h ASFV infection and characterized by hyperdiploid nuclei (Figure-6c). They are determined in the PCBM population until the end of the experiment. In intact, PCBM lymphoblast is represented by cells with di-, hyperdi-, and tetraploid DNA, during the cultivation histogram shifts to the left (Figure-6d). During ASFV infection in lymphoblasts, histogram shifts to the right and hyper tetraploid nucleuses appear (Figure-6e).

### RNA synthesis

**Figure-7:**
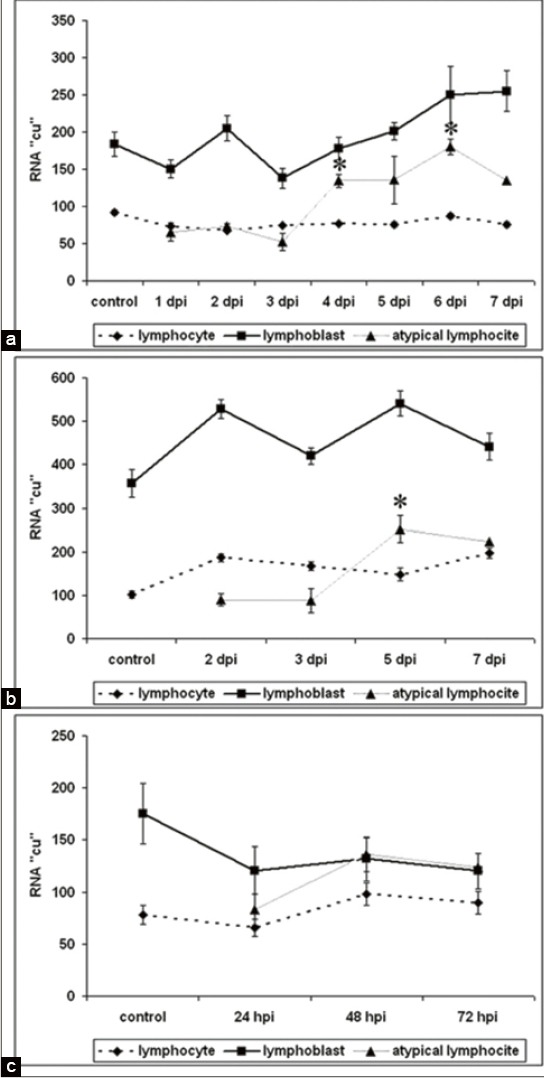
RNA content in lymphoid cells in the peripheral blood, bone marrow (BM) and peripheral blood mononuclear cells (PBMC) at acute African swine fever infection. *Significant compared to lymphocytes (p<0.05-0.01). (a) RNA in blood cells, (b) RNA in BM cells, (c) RNA in PBMC cells.

RNA amount in nucleolus, nucleus, and cytoplasm in all lymphoid cells in blood, BM, and peripheral blood mononuclear cells were examined using cytophotometric procedures after staining with gallocyanin-chromalum staining. The RNA content of peripheral blood lymphoid cells in ASF dynamics are shown in the Figure-7. After the image cytometric measurement of the cellular RNA of each swine peripheral blood lymphoid cells, mean values were compared between ASF and controls.

Measurements of the total RNA in lymphoid cells of all investigated groups showed that it was higher in lymphoblasts compared with both: Lymphocytes and atypical lymphocytes.

We obtained that the RNA content of atypical lymphocyte was lower or similar compared with normal lymphocytes at the beginning of infection. The RNA content in atypical lymphocytes was increased significantly with increasing length of infection and became higher compared to lymphocytes at 4 dpi in blood, at 5 dpi in BM, and at 48 hpi in PCBN.

The gallocyanin-chromalum staining showed decrease in the total RNA level in atypical lymphocytes at early stage of infection *in vivo* conditions (Figure-3) that indicates the general suppression of biosynthetic processes.

### Cytoplasmic ASFV replication complexes

**Figure-8:**
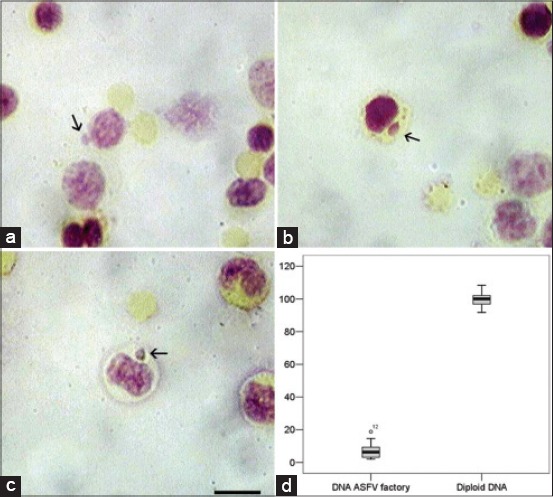
African swine fever virus (ASFV) factories in porcine leukocytes. Arrows show Feulgen positive factories in infected cell cytoplasm, (a and b) Infected lymphocytes, (c) infected monocyte, (d) DNA amount in ASFV factories compared with diploid porcine lymphocytes (%). Y-axis is the percent. The DNA amount in diploid cells is taken as 100%. Scale bare is 10 µm.

ASFV assembles in perinuclear viral factories located close to the microtubule organizing center. It is visible as Feulgen-positive inclusions located in the perinuclear region of infected cells (Figure-8a-c). Measured amount of DNA in the ASFV factories did not exceed 5-8% (with few exceptions) from a diploid cell standard (Figure-8d).

## Discussion

So, atypical lymphocytes morphology includes altered nuclei or/and larger cells with slightly less condensed chromatin and those with a lower N:C ratio. The majority of these cells were CD2 positive. CD2 glycoprotein expressed on all mature human T-cells, thymocytes, most natural killer cells, and a small proportion (9-12%) of BM cells. In swine peripheral blood, “most of the TcR-αβ T-cells express CD2, like CD2+CD4+CD8−, CD2+CD4+CD8^low^, CD2+CD8^low^CD4−, CD2+CD8^high^CD4−, in contrast to TcR-γβ T-cells which are CD2−CD4−CD8−, CD2+CD4−CD8^low^, and CD2+CD8−CD4−; in addition, there is a large proportion of non-T (CD3−), non-B (surface Ig−) lymphocytes expressing CD2, (CD2+CD4−CD8^low^)” [10]. CD2+CD8- cells in swine represent an effector/memory subset, and CD2+CD8+ γβ T-cells probably represent terminally differentiated cells [11]. The majority of detected atypical lymphocytes expressed CD2 T-lymphocyte-associated cell surface antigen, so they belong to TcR-αβ T-cells or non-T, non-B-lymphocytes expressing CD2.

Atypical lymphocytes are known to occur in numerous viral diseases, including infectious mononucleosis, herpes, rubella, influenza, dengue fever, and others. Although the function of atypical lymphocytes is unclear, they are similar in appearance to lymphocytes which undergo blast transformation after stimulation with mitogens (such as phytohemagglutinin) or specific antigens [12].

Formation of atypical lymphocytes during ASFV was shown in *in vitro* conditions (PCBN) [5], and in in vivo conditions in peripheral blood of pigs [4].

There are no standard definitions regarding the morphology of the atypical lymphocyte, and usually, morphological interpretation is based on individual experience [13]. However, Gavosto *et al*. [14] found that a higher than normal proportion of leukocytes synthesized DNA present in the peripheral blood of patients with infectious mononucleosis. After these, investigation analysis of DNA amount became prominent diagnostic criterion in atypical lymphocytes identification. So, investigation in DNA of pathological cells is an important tool for distinguish reactive lymphocytes or virocytes and atypical cells.

It is important to mention that at late stages of cultivation of BM primary culture pathological lymphocytes appears which reminds atypical cells. However, lymphocytes of intact PCBM do not contain DNA exceeding diploid amount [15].

Under the influence of ASFV in PCBM was mentioned not only hyperdiploid lymphocytes but also tetraploid and hyper tetraploid cells [5].

The results confirm earlier observations of a high percentage of cells in DNA synthesis in the peripheral blood of pigs with acute ASF. It may be assumed that the mononuclear cells in DNA synthesis are preparing to cell division. However, in none of the about 100 investigated direct smears, in which at least 10,000 cells were counted were mitoses noted.

Several explanations for this apparent discrepancy are possible (some of postulates were suggested by Epstein, Brecher [16]: (1) The period of DNA synthesis may be very long relative to the period of actual mitosis; (2) the cells may synthesize DNA without ever dividing; (3) the DNA synthesis observed may be that of an intracellular virus rather than of nuclear DNA or an additional amount of DNA in lymphocytes may be identified as a result of accumulations of viral genomes in ASFV factories; (4) the cells may divide only extravascularly possibly because plasma contains some mitosis inhibiting factor or they may circulate only briefly so that the chance of a mitosis occurring in the peripheral blood becomes negligible; (5) the cells may belong to lymphoblasts.

Our observations make the first possibility unlikely (total absence of mitosis).

The second possibility is seems plausible. As shown by Enjuanes *et al.*, [17] after infection with ASFV, there is stimulation of host DNA synthesis and not only in porcine cells but also in chicken macrophages.

The third possibility is unlikely for two reasons. First, ASFV replicates exclusively within the host cell cytoplasm, although a nuclear step has been also reported [18]. Newly synthesized viral DNA is found in the nucleus and cytoplasm in the early stages of DNA replication and is found only in the cytoplasm at later stages. Smaller DNA fragments are synthesized in the nucleus, with larger genome length fragments found in the cytoplasm at later time points [18,19]. This small DNA fragments usually undetectable by cytometry. Virus morphogenesis takes place in virus factories (which can be measured by the cytometry) that are located in the perinuclear zone [20]. Furthermore, atypical lymphocytes cannot be masked by the accumulation of viral DNA. First, viral factories are separate from the cell nucleus and are clearly visible. Second, the amount of viral DNA in ASFV factories is much lower than the increased amount of DNA in atypical lymphocytes.

At present, the fourth possibility that the cells in DNA synthesis divide extravascularly, appears unlikely, as there is no any data about divided lymphocytes in tissues at ASF.

The fifth possibility that the at least part of atypical cells may belong to lymphoblasts is likely for two reasons. First, the majority of atypical cells morphologically resemble (but not similar) lymphoblasts. Second, from the early stages of ASF, immature forms of white blood cells, such as lymphoblasts appeared in the blood of infected pigs [4].

We suggested that ASFV causes a rapid derepression of the lymphocyte genome, increased synthesis of messenger RNA and a consequent increase in the rate of protein synthesis. The results reported here are compatible with non-specific lymphocyte activation such a mechanism of action for phytohemagglutinin [21]. A high rate of RNA synthesis usually might be anticipated in a population of cells with increased metabolic requirements.

Thus, the occurrence of atypical lymphocytes can be an unusual and specific cellular response to ASFV infection. It is also possible that atypical lymphocytes (*in vivo*) represent a response to non-specific viral stimulation or to specific viral antigens due to recognition followed by activation.

## Conclusion

ASFV (genotype II) can cause the formation of atypical lymphocytes *in vivo* and *in vitro* condition. Atypical lymphocytes are identified by their increased size, high metabolic activity, and the presence of additional DNA amount. Furthermore, in atypical lymphocytes, N:C ratio usually decreased, compared to normal lymphocytes. In morphology, they looking like lymphocytes transformed into blasts by exposure to mitogens or antigens *in vitro*. They vary in morphologic detail, but most of them are CD2 positive.

## Authors’ Contributions

ZAK and EMK designed the experiment. LOA, LHH, ASA, and KNY conducted the experiment. ZRT did technical writing and revision of the manuscript. ZAK and EMK prepared the manuscript. All authors have read and approved the final version of the manuscript.
